# Genetic pathways, prevention, and treatment of sporadic colorectal cancer

**DOI:** 10.18632/oncoscience.59

**Published:** 2014-06-30

**Authors:** Rasika Mundade, Thomas F. Imperiale, Lakshmi Prabhu, Patrick J. Loehrer, Tao Lu

**Affiliations:** ^1^ Department of Pharmacology and Toxicology, Indiana University School of Medicine, Indianapolis, IN USA; ^2^ Division of Gastroenterology and Hepatology, Regenstrief Health Center, Roudebush VA Medical Center, Indianapolis, IN USA; ^3^ Division of Hematology and Oncology, Indiana Cancer Pavilion, Indianapolis, IN USA; ^4^ Department of Biochemistry and Molecular Biology, Indiana University School of Medicine, Indianapolis, IN USA

**Keywords:** colon cancer, genetic pathway, sporadic

## Abstract

Epithelial cancer of the colon and rectum, also known as colorectal cancer (CRC), results from a progressive accumulation of genetic and epigenetic alterations that lead to uncontrolled growth of colonocytes, the cells lining the colon and rectum. CRC is the second leading cause of cancer-related deaths and the third most common cancer in men and in women in the U.S. Of all the patients diagnosed with CRC every year, it is estimated that the vast majority of CRCs are non-hereditary “sporadic cancers” with no apparent evidence of an inherited component. Sporadic CRC results from the cumulative effects of multiple genetic and epigenetic alterations caused by somatic mutations, which may themselves be the indirect result of several environmental factors. This review examines our current understanding of the major genetic alterations leading to colon cancer, options for prevention and early detection of CRC, and the currently available treatment approaches that may target these different genetic alterations.

## INTRODUCTION

CRC is a common, heterogeneous disease that arises through the aggregate effects of multiple genetic mutations and epigenetic alterations involving genes that regulate cell growth and differentiation. There are approximately 160,000 new cases of CRC every year in the United States and approximately one-third of CRC patients die from the disease [1]. In the United States, the lifetime risk of developing CRC for both men and women is 6% and the average age at diagnosis is 66 years [2]. Though there has been considerable advancement in the management of CRC, mortality remains high and unchanged with the 5-year survival rate of only 62%, which is attributable largely to complications of metastatic disease [2].

CRC presents with a broad spectrum of neoplasms, ranging from benign growths to invasive cancer. CRC starts in the inner lining of the colon and/or rectum as a growth of tissue called a polyp slowly growing through some or all of its layers. A particular type of polyp called the adenomatous polyp or adenoma is a benign tumor that may undergo malignant transformation to cancer. This malignant transformation is the result of mutation or deletion of major regulator genes, resulting first in hyperplasia moving toward adenoma to carcinoma and then metastasis [3].

A present estimate is that between 15–30% of CRCs may have a major hereditary component, given the occurrence of CRC in first- or second-degree relatives [4]. Most of the colorectal heritable syndromes are attributable to either familial adenomatous polyposis (FAP) or hereditary nonpolyposis colorectal cancer (HNPCC) [5]. It is important to note, however, that most cases of CRCs (70–85%) are “sporadic” and the patients have no identifiable genetic risk factors. The development of sporadic colon cancer is thought to be influenced by diet, lifestyle, environmental factors, and acquired somatic mutations [6]. The spectrum of somatic mutations contributing to the pathogenesis of CRC is likely to be far more extensive than previously appreciated. Thus elucidating the underlying genetic pathways in the genesis of CRC would provide fertile ground for basic research and may also lead to potential prognostic information and targets for novel therapies.

### Genetic pathways of sporadic colon cancer

**Figure 1:**
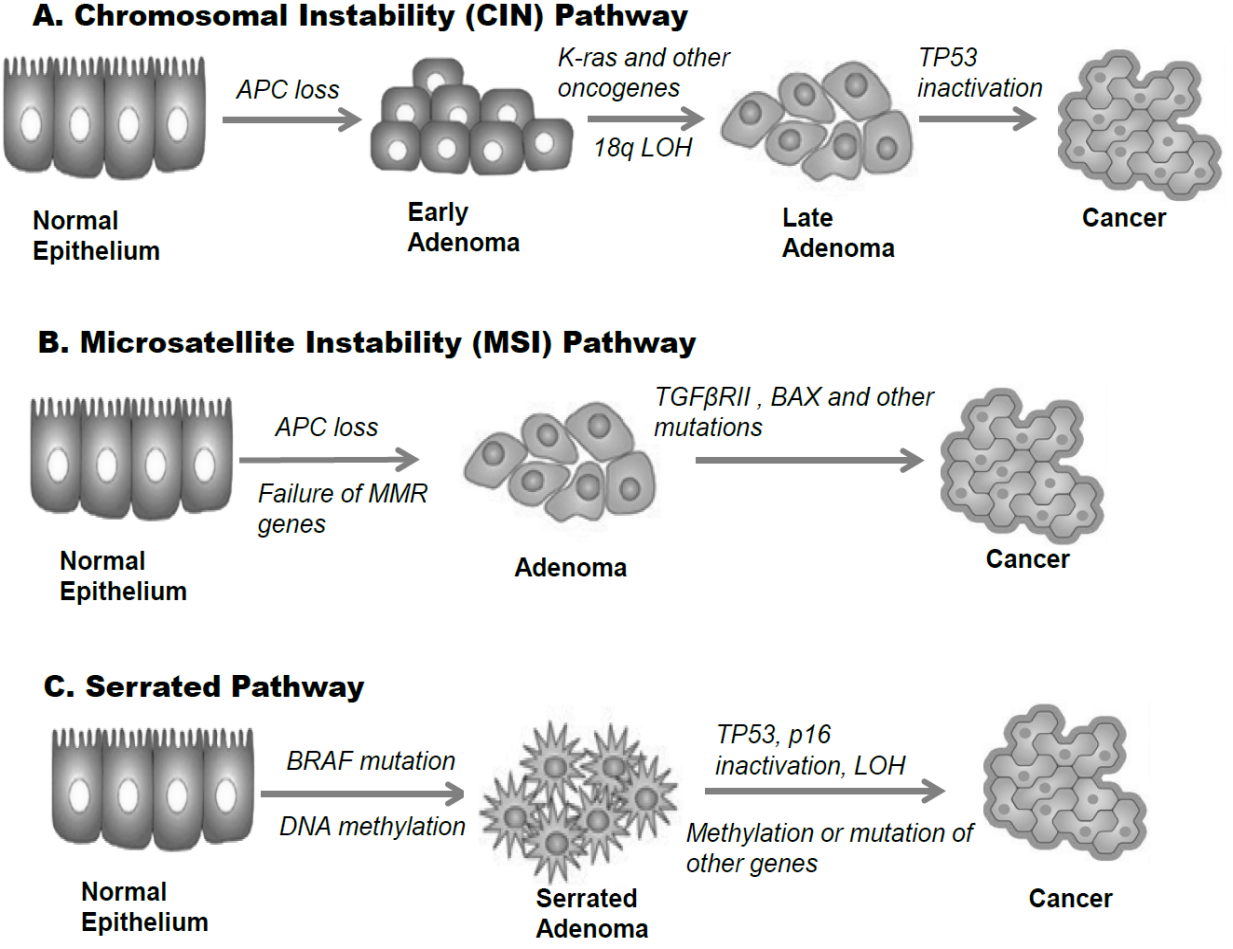
Multiple genetic pathways in colorectal cancer pathogenesis Three distinct parallel pathways are implicated in CRC pathogenesis: Chromosomal Instability (CIN), Microsomal Instability (MSI), and Serrated Pathway. The sequential genetic and epigenetic changes occurring in each pathway are simplified. (A) The CIN pathway is driven by inactivating mutations in tumor suppressor genes, such as the adenomatous polyposis coli (APC) gene and activating mutations in proto-oncogenes, such as KRAS etc. which lead to increased clonal expansion of the cells. Subsequent loss of heterozygosity (LOH) for the long arm of chromosome 18 (18q) and loss of tumor suppressor TP53 confer these expanding cells with additional growth advantages which ultimately leads to invasive cancers. (B) The MSI pathway is driven by the loss of APC gene, and is characterized by inactivation of the mismatch repair (MMR) genes, such as MutL homolog 1 (MLH1) *etc.* The inactivation of MMR genes mostly is caused by epigenetic silencing via promoter hypermethylation. The failure of MMR genes subsequently leads to mutations in specific target genes involved in proliferation and cellular differentiation such as transforming growth factor β receptor II (TGFβRII); proteins involved in apoptosis regulation such as BAX and others, ultimately leading to microsatellite unstable invasive tumors. (C) The Serrated Pathway is driven by hypermethylation of genes and is characterized by presence of proto-oncogene BRAF mutation which causes increased MAPKs/ERKs signaling, leading to increased cell proliferation. Subsequent methylation of other genes and loss of tumor suppressor genes such as TP53, p16 *etc.* will lead to CRC.

It is well established that sporadic CRC is a genetic disease caused by sequential accumulation of mutations in multiple genes. Over the past three decades, molecular genetic studies have identified several crucial gene defects that underlie predisposition to sporadic CRC [5]. As shown in Figure 1, there are three major genetic mechanisms responsible for sporadic CRC, namely chromosomal instability (CIN); microsatellite instability (MSI) and the CpG island methylator phenotype (CIMP) pathways [7]. The majority of sporadic CRCs are due to events that result from aberrations in the CIN pathway [8].

### CIN pathway

The CIN pathway is an adenoma-carcinoma sequence model (Fig. 1A) which suggests that a stepwise pattern of mutational activation of oncogenes and inactivation of tumor suppressor genes result in CRC [9]. The genomic changes include activation of proto-oncogenes KRAS, c-Src, c-Myc and inactivation of at least three tumor suppressor genes, such as the loss of Adenomatous polyposis coli (APC) gene, tumor suppressor p53 (TP53) gene, and heterozygosity for the long arm of chromosome 18 (18q LOH) [10, 11].

Among the earliest events in sporadic CRC progression pathway is the loss of the APC gene. Genetic disruption of the APC gene or its inactivation by hypermethylation of the APC promoter leads to Wnt/β- catenin signaling activation. This process is hypothesized to be the key event for adenoma initiation [12, 13]. Mutant APC protein increases stabilization of β-catenin and leads to its accumulation in the cytoplasm and its eventual translocation into the nucleus to act as a co-activator of the T-cell factor/lymphoid enhancer factor family (TCF/LEF) transcription factors. This process in turn will activate a repertoire of genes that are involved in cell proliferation and growth [14]. The importance of Wnt/β-catenin signaling in the genesis of CRC is further reflected in many CRCs (50%) with intact APC genes but high frequency of activating mutations in β-catenin that harbors functionally significant phosphorylation sites [15].

Another important genetic pathway contributing to CIN is KRAS. The KRAS gene belongs to the RAS family of oncogenes and is mutated in 30–50% of CRCs [16]. RAS proteins play important roles in cell division, cell differentiation, and apoptosis. Multiple cellular functions are regulated by activated RAS through different pathways. One of the best characterized pathways regulated by RAS family is the Raf–mitogen-activated protein kinase kinase (MEK)–extracellular signal-regulated kinase (ERK) pathway, which is involved in the control of cell cycle progression [17]. Mutation in KRAS disrupts the RAS signaling pathway leading to tumorigenesis. These mutations impair the intrinsic Guanosine-5′-triphosphate (GTP)ase activity of KRAS, allowing KRAS to accumulate in the active, GTP-bound conformation and lead to constitutive activation of its downstream pro-proliferative signaling pathways [18].

Studies have also demonstrated the loss of tumor suppressor TP53 gene and 18q LOH as major contributors to the CIN phenotype [19]. The TP53 gene is significantly involved in the control of the cell cycle and apoptosis and is commonly mutated in CRC, leading to uninhibited cell growth [9]. SMAD4, an important tumor suppressor present in chromosome 18q21.1 is lost by 18q deletion, resulting in tumorigenesis via the transforming growth factor β (TGFβ) pathway [20]. The presence of 18q LOH has been proposed as a worse prognostic marker for patient survival in CRC [21]. Recently, mutations in genes of phosphatidylinositol 4,5-bisphosphate 3-kinase catalytic subunit α (PIK3CA) and TGFβ receptor (TGFBR) also have been shown to play a role in CRC development [22].

### MSI pathway

In addition to CIN pathway, about 10–15% of sporadic CRC are due to the MSI pathway (Fig. 1 B). MSI is the condition of genetic hypermutability that results from impaired DNA mismatch repair (MMR). In other words, MSI is the phenotypic evidence that MMR isn't functioning normally. The proteins involved in MMR form a complex that binds to the mismatch, identifies the correct strand of DNA, then subsequently excises the error and repairs the mismatch. Cells with abnormally functioning MMR tend to accumulate mutations (insertions or deletions) in microsatellites located in DNA coding regions, generating frameshift mutations and subsequently leading to sporadic CRCs [23].

Inactivation of MMR genes occur either through aberrant methylation of promoter CpG of the MutL homolog 1 (MLH1) gene or point mutations in one of the MMR genes, with the former reason accounting for most of the cases of MMR inactivation [22]. As a result of defects in the DNA MMR system, MSI cancers more readily acquire mutations in important cancer-associated genes as compared to cells that have an effective DNA MMR system. Among the earliest events in the MSI-dependent CRC progression, similar to the CIN pathway (Fig. 1A), loss of function of the APC gene product (Fig. 1B) also appears to play an important role in predisposing persons with germ line APC mutations to sporadic CRCs [22].

Clinically, solely based on the extent of MSI, CRCs can be classified as MSI-high, MSI-low, or microsatellite stable (MSS) [23]. As compared with MSS/MSI-low tumors, MSI-high tumors form a well-defined group with distinct clinicopathological features. This type of CRC often arises from the epigenetic silencing of the MMR gene, such as MLH1 (Fig. 1B), so it belongs to the traditional MSI pathway. In contrast, MSS/MSI-low tumors arise through CIN pathway (Fig.1A). Overall, MSI-high tumors have a better long-term prognosis than MSI-low/MSS tumors. In general, MSI and CIN CRC respond differently to chemotherapeutics and have implications for specialized management of patients [24, 25].

### Serrated pathway

The name of this pathway is attributable to the morphologically serrated appearance of the precursor lesions. Different from the CIN (Fig.1A) and MSI (Fig.1B) pathways, in which the sporadic CRCs are mainly initiated through classical APC mutations, the Serrated pathway initiated CRCs are highlighted by the presence of BRAF (protein kinase B-Raf) mutation and epigenetic silencing of genes that are involved in cell differentiation, DNA repair, and cell-cycle control, but not APC (Fig. 1C) [26, 27].

BRAF, a member of the RAF kinase family is a serine/threonine-specific protein kinase that plays a key role in regulating the MAPKs/ERKs (mitogen-activated protein kinases/extracellular signal-regulated kinases) signaling pathway, which affects cell division, differentiation, and secretion. Point mutation in BRAF (V600E) causes constitutive activation of this kinase as well as its insensitivity to negative feedback mechanisms, leading to enhanced MAPK/ERK signaling [28]. This overactive signaling cascade reaches cellular DNA within the nucleus and triggers downstream effectors to induce uncontrolled cell proliferation, evasion of immune response, angiogenesis [through MAPK-dependent activation of hypoxia-inducible factor 1 α (HIF-1α), vascular endothelial growth factor (VEGF)], tissue invasion, and metastasis (via upregulation of several proteins involved in migration, integrin signaling, and cell contractility), as well as resistance to apoptosis [29].

Among the epigenetically silenced genes in Serrated pathway, p16 (also known as cyclin-dependent kinase inhibitor 2A, multiple tumor suppressor 1) is one of the most well- characterized tumor suppressor genes. The p16 tumor suppressor protein functions as an inhibitor of CDK4 and 6 (cyclin-dependent kinase 4 and 6), the D-type cyclin-dependent kinases that initiate the phosphorylation of the retinoblastoma (Rb) tumor suppressor protein. The progression of sporadic CRCs through the Serrated pathway is accelerated by p16 inactivation through promoter hypermethylation.

It is possible that no two CRCs are alike and only a few mutations are common to most sporadic CRCs. Therefore, each tumor has its own unique combination of genetic alterations. In addition to the pathways described, the heterogeneity of CRCs is further attributed to the interactions of the described pathways (Fig.1) with other less described or still undescribed pathways. An example can be seen in the Landscaper Defect pathway, in which the defective cells are derived from the stroma and epithelial tumorigenesis is the result of an abnormal microenvironment [30]. These different pathways will undoubtedly interact with each other, and may even modify these routes to carcinogenesis.

### Prevention

Although the incidence and mortality rates from CRC are declining steadily in the United States, health disparities in cancer screening, treatment, and survival still persist [31]. Because CRC and most adenomatous polyps are usually asymptomatic during the early stages, screening is critical to reducing morbidity and mortality. Over the past two decades, screening has contributed to a significant decline in both the number of CRC cases and the number of CRC deaths. While several strategies are recommended by the three major guideline organizations [32-34] (Table 1), the mainstay of screening involves fecal occult blood testing with either high-sensitivity guaiac-based fecal occult blood tests (FOBT) or fecal immunochemical tests (FIT), and bowel examination by lower endoscopy. Colonoscopy is the most widely used screening test used in the U.S. [35]. While it has the highest diagnostic sensitivity and specificity of all available tests, it is uncertain whether a strategy of colonoscopy every ten years is best. It is possible that a less sensitive test that is frequently applied may be as effective as colonoscopy, as suggested by simulation models [36-40]. Such uncertainty and resulting equipoise constitute the basis for two ongoing randomized trials of colonoscopy versus FIT being conducted in Spain [41] and by the Veterans Affairs in the U.S. [42], the results of which will be available in ten years or so. Recently, stool DNA testing has been shown to be more sensitive than FIT for both CRC and advanced, precancerous polyps [43] and may be an effective non-invasive alternative to annual FIT. CRC screening recommendations by all three guideline organizations are expected to be updated within 1–2 years.

**Table 1:** Average-Risk Screening Tests and Intervals

	Guideline Organization		
Test	American Cancer Society- Multi-society Task Force- American College of Radiology [32]	United States Preventive Services Task Force [33]	American College of Gastroenterology [34]
Guaiac-based fecal occult blood test (gFOBT)	Not recommended	Annually	Not recommended
High sensitivity gFOBT or fecal immunochemical test (FIT)	Annually	Annually	Annually
Sigmoidoscopy	Every 5 years	Every 5 years (suboptimal)	Every 5 years
High sensitivity gFOBT or FIT and sigmoidoscopy	Every 1 and 5 years, respectively	Mid-interval and every 5 years, respectively	Every 1 and 5-10 years, respectively
Double contrast barium enema	Every 5 years	Not recommended	Not recommended
Fecal DNA	Yes, interval not given	Not recommended	Every 3 years
Computed tomographic colonography	Every 5 years	Not recommended	Every 5 years
Colonoscopy	Every 10 years	Every 10 years	Every 10 years

### Treatment

Depending on the stage and progression state of the disease, treatment regimens for CRCs include: colectomy (Stage 0, Stage I and early Stage II colon cancers), postoperative adjuvant chemotherapy (Stage III and some Stage II colon cancers), chemotherapy with multi-drug therapy including 5-fluorouracil and leucovorin and CapeOx (capecitabine and oxaliplatin) (Stage II) and radiation therapy (recurrent or advanced disease). Another chemotherapeutic agent, imnotecan (CPT- 11) has been shown to improve efficacy in CRCs [44]. Recently, oxaliplatin has been shown to induce immune thrombocytopenia in a CRC patient [45].

Genetically engineered monoclonal antibodies are used in treating CRCs. Cetuximab and panitumumab are anti-EGFR monoclonal antibodies that block the EGFR signaling pathway. These two drugs are used in the treatment of metastatic CRC in combination with conventional chemotherapy or as single agent. Clinical studies have shown that combination therapy with irinotecan plus cetuximab increases the survival rates and response in metastatic CRC patients as compared to irinotecan alone [22]. Recently, the U.S. Food and Drug Administration (FDA) approved bevacizumab for treatment of CRC. Bevacizumab is a recombinant humanized monoclonal antibody that binds to human VEGF, thereby preventing the interaction of VEGF with its receptors. Stivarga (regorafenib), a multi-kinase inhibitor, is another drug that has been approved by FDA for the treatment of metastatic CRC that has continued to spread after treatment. Evaluation of recombinant vaccines for colon cancer has begun with concurrent technologies in the fields of molecular biology and immunology. Very recently, Ye *et al* [46] showed that recombinant salmonella-based 4-1BBL vaccine enhances T cell immunity and inhibits the development of CRC in rats. Research in the field to target alternative pathways such as the anti-apoptotic signaling pathways including NF-κB, Bcl-2, and the TRAIL receptor to treat CRC is still ongoing [47].

The Genetic Sequencing technique has revolutionized monitoring of disease progression, relapse, and residual disease in CRCs [22]. Bass *et al* [48] reported whole genome sequencing from CRC patients. This study led to deeper understanding of the essential gene fusions and other oncogenic events in CRC. Leary *et al* [49] provided a massive parallel sequencing using the PARE (personalized analysis of rearranged ends) approach for the development of personalized biomarkers to enhance the clinical management of CRC patients.

The pace of recent advances in our understanding of the molecular basis of CRC and expansion in the drugs designed to treat CRC have led to substantial gains in quality of life in CRC patients. However, the significant burden of CRC on public health still remains. Like most other cancer therapeutics, these treatment regimens are associated with side effects and have not yet shown significant efficacy in most instances. Another key factor that limits progress in CRC chemoprevention is the pace of clinical research. There is a significant lack of awareness among people to undergo CRC screening despite established and available techniques. Overcoming these challenges will bring cause for optimism and room for hope in treating CRCs.

## Abbreviations

APC: adenomatous polyposis coli gene
Bcl-2: B-cell lymphoma 2
BRAF: protein kinase B-Raf
CDK4: cyclin-dependent kinase 4
CDK6: cyclin-dependent kinase 6
c-Myc v-myc: avian myelocytomatosis viral oncogene homolog
CIMP: CpG island methylator phenotype
CIN: chromosomal instability
CRC: colorectal cancer
c-Src: proto-oncogene tyrosine-protein kinase Src
DNA: deoxyribonucleic acid
18q LOH: loss of long arm of chromosome 18
EGFR: epidermal growth factor receptor
ERK: extracellular signal-regulated kinases
FAP: familial adenomatous polyposis
FDA: Food and Drug Administration
FIT: fecal immunochemical test
FOBT: fecal occult blood test
GTP: guanosine-5′- triphosphate
HIF-1α: hypoxia-inducible factor 1 α
HNPCC: hereditary nonpolyposis colorectal cancer
KRAS: Kirsten Rat Sarcoma Viral Oncogene Homolog
LEF: lymphoid enhancer factor
MAPK: mitogen-activated protein kinase
MEK: mitogen-activated protein kinase kinase
MLH1: mutL homolog 1
MMR: mismatch repair
MSI: microsatellite instability
MSS: microsatellite stable
NF-κB: nuclear factor κB
PARE: personalized analysis of rearranged ends
PIK3CA: phosphatidylinositol 4,5-bisphosphate 3-kinase catalytic subunit α
Rb: retinoblastoma
Raf: rapidly accelerated fibrosarcoma
Ras: rat sarcoma
SMAD4: mothers against decapentaplegic homolog 4
TCF: T-cell factor
TGFβ: transforming growth factor β
TGFβR: transforming growth factor β receptor
TP53: tumor suppressor p53 gene
TRAIL: TNF-related apoptosis-inducing ligand
VEGF: vascular endothelial growth factor
Wnt: wingless
